# Alternative procedure to shorten rectal barostat procedure for the assessment of rectal compliance and visceral perception: a feasibility study

**DOI:** 10.1007/s00535-012-0543-x

**Published:** 2012-02-24

**Authors:** S. A. L. W. Vanhoutvin, F. J. Troost, T. O. C. Kilkens, P. J. Lindsey, D. M. A. E. Jonkers, K. Venema, A. Masclee, R-J. M. Brummer

**Affiliations:** 1 TI Food and Nutrition, Wageningen, The Netherlands; 2 Division of Gastroenterology-Hepatology, Department of Internal Medicine, NUTRIM (UNS50, box 46), Maastricht University Medical Center, P.O. Box 616, 6200 MD Maastricht, The Netherlands; 3 Mondriaan Zorggroep, Heerlen, The Netherlands; 4 Department of Population Genetics, Genomics and Bioinformatics, Maastricht University, Maastricht, The Netherlands; 5 Department of Biosciences, TNO Quality of Life, Zeist, The Netherlands; 6 School of Health and Medical Sciences, Örebro University, Örebro, Sweden

**Keywords:** Barostat, Humans, Rectal, Visceral perception, Rectal compliance, Rectal capacity, Minimal distension pressure, Irritable bowel syndrome

## Abstract

**Background:**

Barostat methodology is widely used for assessing visceral perception. Different barostat protocols are described with respect to the measurement of rectal compliance and visceral perception. The choice of protocols affects the duration, which is normally 60–90 min, and accuracy of the procedure. This study aimed to shorten the procedure by using the semi-random distension protocol for both compliance and visceral perception measurement and a correction based on rectal capacity (RC) instead of minimal distension pressure (MDP).

**Methods:**

Twelve irritable bowel syndrome (IBS) patients (7 females) and 11 healthy controls (8 females) underwent a barostat procedure. Compliance was determined during both a staircase distension and a semi-random protocol. Visceral perception data were compared as a function of pressure or relative volume, corrected for MDP or RC, respectively.

**Results:**

Compliance measurement using the semi-random protocol instead of the staircase distension protocol resulted in an overestimation in healthy volunteers, but not in IBS patients. The overall conclusion that IBS patients had a lower compliance compared to controls was not different between protocols. Data presentation of the visceral perception scores as a function of corrected volume instead of pressures corrected for MDP did not alter the conclusion that sensation scores in IBS patients were higher as compared to healthy controls.

**Conclusions:**

This study showed that barostat procedures may be shortened by approximately 20 min, without losing the ability to discriminate between healthy controls and IBS patients. A correction for RC instead of MDP may improve the accuracy of the procedure.

**Electronic supplementary material:**

The online version of this article (doi:10.1007/s00535-012-0543-x) contains supplementary material, which is available to authorized users.

## Introduction

Alterations in visceral perception and rectal compliance have been observed in several functional gastrointestinal disorders, but the underlying pathophysiological mechanisms are still poorly understood. Several studies demonstrated a decreased rectal compliance and increased rectal sensitivity in patients with irritable bowel syndrome (IBS), compared to healthy controls [1–7]. Visceral perception is generally measured in vivo using the barostat technique. Since its introduction, different distension protocols have been used and efforts have been made to optimise the distension protocols [8–10]. Whitehead and Delvaux [11] described a number of basic recommendations for the measurement of visceral perception and compliance. These recommendations include the use of a thin plastic polyethylene bag instead of a latex balloon, inflation speed, catheter construction in terms of minimal luminal cross sections and pressure monitoring inside the balloon, the use of visual analogue scales (VAS) and the influence of body posture and position during the measurements [11]. In addition, recommendations were given with respect to the distension protocol for determination of compliance, visceral perception, determination of minimal distension pressure (MDP) and first sensation (FS). However, barostat procedures applied for clinical diagnostic purposes and for scientific studies still have different protocols. This hampers comparisons between studies. Some but not all research groups present sensation scores (pain, urge and discomfort) as a function of balloon pressure [12–15], whereas others relate it to balloon volume [9, 14]. In order to correct for inter-individual variation, a correction for MDP and/or rectal capacity (RC) is used by some, but not by others. Moreover, the protocols used to determine MDP and RC differ.

To enable the comparison of results obtained in different studies, initiatives should be taken to come to a generally accepted protocol with standardised cut-offs for RC and/or MDP. Consensus should be achieved with respect to the pressure at which RC should be determined. MDP is defined by some investigators as the pressure at which respiratory waves appear for the first time in the volume curve [9, 13], whereas others define it as the pressure needed to reach a specific volume (e.g. pressure at which the volume reaches 25 ml) [4, 10]. Determination of the different parameters in one barostat procedure requires multiple consecutive distension protocols. Shortening the procedure by combining the determination of several parameters in one distension protocol would provide a major advantage for its use in a clinical setting, because duration of the procedure has important implications for patient burden as well as for total costs.

The primary aim of this study was to shorten the barostat procedure by using the semi-random distension protocol for both compliance and visceral perception measurement, while preserving the ability to discriminate between healthy volunteers and IBS patients. This would shorten the duration of the barostat protocol and, hence, lower the patient and labour burden.

## Methods

### Subjects

Twelve IBS patients (based on Rome III criteria; 7 females, mean age 42 ± 14 years) and 11 healthy controls (8 females, mean age 33 ± 15 years) were included in this study. Five of the IBS patients had diarrhea-predominant IBS, 5 suffered from constipation-predominant IBS, and 2 patients had the alternating type. No differences were found on the basis of age or gender between both groups. Body mass index (BMI; kg/m^2^) did not significantly differ between IBS patients (mean 24, CI 22.5–25.5) and healthy volunteers (mean 23.9, CI 22.1–25.8). None of the volunteers had a history of abdominal surgery. No medication was allowed during the study unless subjects were on stable medication for at least 3 months prior to and during the study. The study was approved by the Medical Ethics Committee of University Hospital Maastricht and conducted in accordance with the principles of the Declaration of Helsinki (52nd WMA General Assembly, Edinburgh, Scotland, Oct 2000). All volunteers gave their written informed consent prior to participation. Baseline data from two interventional studies (http://www.clinicaltrials.gov, NCT00696098 and NCT00726817) were used for the present study. All subjects participated in a single barostat measurement.

### Barostat protocol

All subjects underwent the same barostat procedure as described before [13]. After an overnight fast, the subjects arrived in the hospital and self-administered a rectal enema containing 60 ml of saline to clean the rectum. Five minutes thereafter, patients were instructed to void rectal contents.

Subsequently, the patients laid down on a bed in a left lateral supine position and remained in this position during the entire test procedure. This position was chosen to minimize the intra-abdominal pressure. A commercially available barostat balloon (Mui Scientific C7-2CB-R, ON, Canada) was lubricated with KY gel (Johnson & Johnson, Langhorne, Pennsylvania) and inserted rectally 3 cm proximal to the anal sphincter. The balloon had a volume of 500 ml and was made of PVC. After a 5-min habituation period, the balloon was attached to the barostat equipment (Distender II, G&J Electronics, ON, Canada) and the barostat procedure was started. The controlled balloon distensions were programmed using the standard software package of the barostat equipment (Protocol Plus Deluxe, version 6_7; G&J Electronics, ON, Canada).

**Fig. 1:**
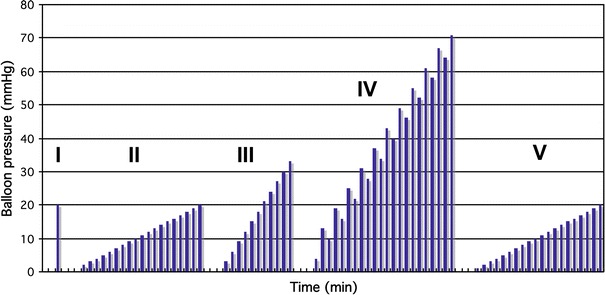
Barostat protocol that was applied in this study. It contained 5 consecutive distension protocols (I–V). Protocol I was designed for balloon unfolding, protocol II for determination of minimal distension pressure (MDP-1) and first sensation (FS-1), protocol III for compliance 1 and RC measurement, protocol IV for visceral perception and compliance 2, and protocol V for the assessment of MDP-2 and FS-2

The barostat protocol consisted of five sub-protocols, each designed for the measurement of specific parameters of interest (Fig. 1). The total duration of the barostat procedure was 60–90 min. After inclusion, prior to the start of the study, all subjects underwent a dummy barostat procedure, which consisted of a reduced number of distensions of different intensities. During this dummy barostat procedure, subjects were to get familiar with the barostat technique and the VAS scores in order to reduce the amount of fear and anxiety on the day of testing.

#### Protocol I: balloon unfolding

The first part of the protocol consisted of a single distension at a balloon pressure of 20 mmHg for 1 min, to ensure that the balloon was placed correctly without folds that may impair airflow.

#### Protocols II and V: minimal distension pressure

The second part of the protocol consisted of a staircase distension protocol with pressure steps of 1 mmHg with a duration of 30 s each and a range from 0 to 20 mmHg. The MDP, which is the minimal balloon pressure required to overcome the intra-abdominal pressure, was defined as the first pressure at which respiratory curves were present in the volume recording of the balloon. The entire protocol was performed up to the 20 mmHg pressure in all subjects and served as a sensitisation step prior to the compliance and perception measurements. The obtained MDP value was set to zero as a reference point during the measurement of visceral perception (protocol IV). During this protocol the patients were asked to report the moment at which they sensed the balloon for the first time. This pressure was defined as the threshold for FS. The measurements of MDP and FS were repeated at the end of the protocol (protocol V) to check the stability of these variables during the barostat procedure.

#### Protocol III: compliance and rectal capacity

Directly after finishing the MDP and FS measurements, the third part of the protocol was initiated. This part of the protocol, designed for determining compliance, consisted of a staircase distension protocol with pressure steps of 3 mmHg with a duration of 30 s each and a pressure range of 0–33 mmHg. Pressure–volume curves from both the staircase distension (part III of the protocol, i.e. compliance 1) and the semi-random distension (part IV of the protocol, i.e. compliance 2) were used to compare the compliance measurements. Dynamic compliance was defined as the slope of the pressure–volume curve at the steepest part (at the inflection point of the curve). In addition, RC, which was defined as the volume at a pressure of 33 mmHg, was determined. RC was used to correct the measured volumes for differences in individual RC. Consequently, all volumes are expressed as a percentage of the individual RC (=index volume).

#### Protocol IV: visceral perception

Subsequently, the distension protocol of the visceral perception measurements was initiated. This protocol consisted of semi-random distensions (at 4, 13, 10, 19, 16, 25, 22, 31, 28, 37, 34, 43, 40, 49, 46, 55, 52, 61, 58, 67, 64, 71 mmHg above MDP, respectively) with a duration of 1 min each, interspaced with 30-s intervals at MDP. Thirty seconds after the start of each distension, patients scored the sensation of pain and discomfort on a 10-cm VAS and urge on a 6-point scale (0, no feeling; 1, just sensible; 2, clearly sensible/light urge; 3, normal urge; 4, strong urge/have to run to toilet; 5, maximum/stop) represented by 6 buttons on an electronic control panel (Distender II perception panel), which was directly linked to the barostat equipment. The procedure was stopped when the maximum score for pain, urge or discomfort was reached.

### Statistical analysis

#### Minimal distension pressure and first sensation data analysis

MDP and FS were each analysed using a Gaussian linear regression. For both analyses, the BMI, FS and compliance were included during model building. The inference criterion used for comparing the models is their ability to predict the observed data, i.e. models are compared directly through their minimized minus log-likelihood. When the numbers of parameters in models differed, they were penalized by adding the number of estimated parameters, a form of the Akaike information criterion (AIC) [16]. For each variable of interest, the group was then added to the model. The effects were considered significant if the AIC decreased compared to the model not containing the group.

MDP, BMI and RC were also analysed by pairs using a bivariate Gaussian linear regression including the appropriate covariance structure in order to capture the dependence between them. The compliance and FS were included as explanatory variables during model building. The AIC was used to assess whether there was a group effect.

#### Rectal capacity data analysis

The RC volume was analysed using a Gaussian non-linear regression including the pressure and compliance as explanatory variables. The AIC was used to assess whether there was a group effect.

#### Visceral perception data analysis

The pain and discomfort data were analysed using a multivariate Gaussian non-linear regression including, if necessary, a random effect and a first-order autocorrelation. Urge was scored on an ordinal 6-point scale and was analysed using a mixture of a logistic distribution (parameterized as a proportional odds) and a gamma distribution (to introduce frailty and autocorrelation dependencies) [17]. The mean regression was imposed through the pressure variable to follow a logistic (‘S-shape’) curve. The model included MDP and FS as explanatory variables. As for the other analysis, the AIC was used to assess whether there was a group effect.

A more detailed description of the analyses is provided in the supplementary material (S1).

## Results

### Visceral perception

**Fig. 2:**
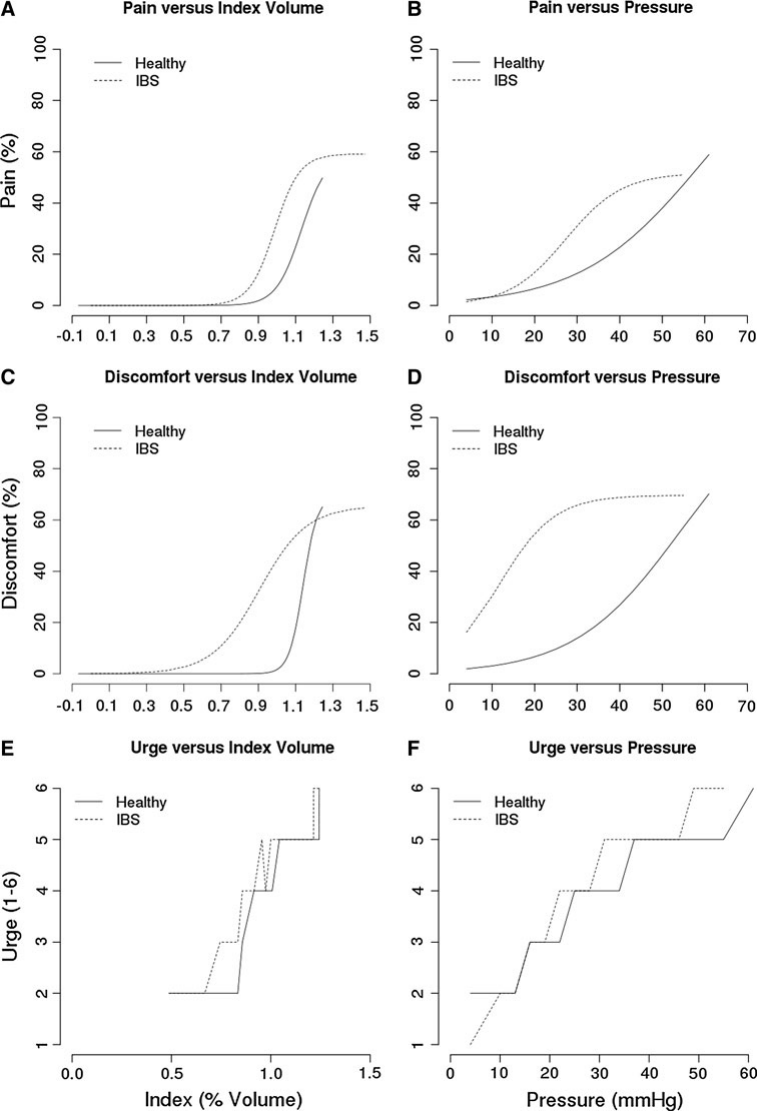
Perception scores for pain, discomfort and urge presented as either a function of index volume (**a**, **c**, **e**, respectively) or as a function of pressure (**b**, **d**, **f**, respectively) in IBS patients and in healthy controls. In all cases, IBS patients showed higher sensation scores compared to healthy controls

Figure 2a–f show the perception scores for pain, urge and discomfort presented as a function of pressure (corrected for individual differences in MDP) and as a function of index volume (corrected for individual differences in RC). As shown in Fig. 2a, the index volume at which a moderate pain level of 50% is reached is 1.11 and 1.24 for IBS patients and healthy controls, respectively. The confidence intervals for the pain scores at the level of index volume are 44.15–54.97 and 42.34–54.32 for IBS patients and healthy controls, respectively. The individual curves for the two conditions differ significantly. In all cases, IBS patients showed higher sensation scores compared to the healthy controls independent of the presentation of pressure or index volume curves.

#### MDP and FS

MDP and FS were determined in the beginning (1) and at the end (2) of the protocol. No significant differences were detected between MDP-1 (mean 4.9, CI 4.1–5.7) and MDP-2 (mean 5.3, CI 4.6–6.1) and between FS-1 (mean 12.1, CI 10.5–13.7) and FS-2 (mean 11.9, CI 10.3–13.5).

**Fig. 3:**
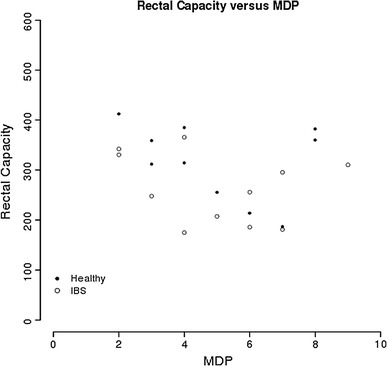
Individual measurements (two missing values) of RC and MDP. No significant correlation was found

IBS patients had a lower FS as compared to healthy controls (mean 6.81 mmHg, CI 5.14–8.74 and mean 12 mmHg, CI 10.73–13.63, respectively). No significant correlation was found between MDP and RC (Fig. 3): RC = 337.4–9.6 × MDP with a confidence interval of the coefficient of −20.2 to 1.0 indicating that the correlation is not significant. No significant correlations were found between BMI and MDP or between BMI and RC (data not shown).

#### RC

RC was determined as the volume at a pressure of 33 mmHg and was used to plot the sensation scores as a function of index volume (volume % of individual RC). RC was not significantly different between healthy volunteers (mean 1.1, CI 1.0–1.2) and IBS patients (mean 1.1, CI 1.1–1.2).

#### Compliance

**Fig. 4:**
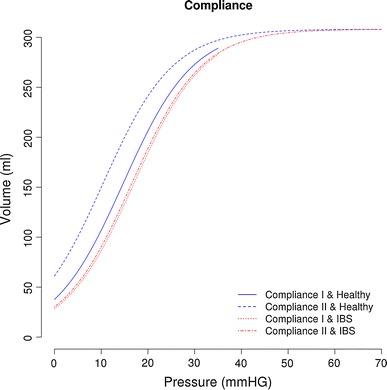
Compliance curves for healthy controls and IBS patients, both calculated in the staircase distension protocol (compliance 1) and in the semi-random protocol (compliance 2)

Within IBS patients, no difference was found between the compliance calculated in parts III and IV of the protocol (compliance 1 and 2, respectively). In healthy controls, calculation of the compliance in the semi-random protocol (compliance 2) resulted in a higher compliance (Fig. 4). Regardless of the protocol chosen, the compliance was significantly lower in IBS patients compared to healthy controls. In addition to a comparison of the overall pressure–volume curves, dynamic compliance was calculated at the inflection point of the pressure–volume curves from Fig. 4. The means and confidence intervals for the dynamic compliance 1 and 2 for the healthy controls were 156.86 ml/mmHg, CI 155.6–158.12 and 199.89 ml/mmHg, CI 198.75–201.04, respectively, and those for the IBS patients were 133.3 ml/mmHg, CI 132.06–134.54 and 137.81 ml/mmHg, CI 136.56–139.05, respectively.

## Discussion

Our data indicate that compliance can be measured in the semi-random protocol instead of the staircase distension protocol, without losing the ability to discriminate between healthy controls and IBS patients. Furthermore, measurements of MDP and FS did not change during the barostat procedure. The visceral perception data expressed as percentage of RC show the same results as those based on balloon pressure, although the presentation of the data differs. Both sets of data lead to the conclusion that perception scores are higher in IBS patients compared to controls. Baseline data from two interventional studies using the same procedure were used for the present study. This led to two highly comparable datasets but also resulted in a lack of perception data in the staircase distension protocol for evaluation of the possibility of measuring multiple parameters in the staircase protocol. The number of patients tested for this study did not allow sub-group analysis of different types of IBS patients.

In the literature, various methods are applied to determine rectal compliance from a pressure–volume curve [8–10]. Both the total fit and the dynamic compliance, which is the slope of the pressure–volume curve at its steepest point, are commonly used techniques to evaluate the compliance. The total fit of the curve provides more information on the pressure–volume relationship at each pressure level without losing statistical power due to multiple testing. In the present study, a total fit of the curve was calculated to evaluate the differences between IBS patients and healthy controls using two different distension protocols (i.e. semi-random vs. staircase). In addition, the compliance values at the steepest point of the pressure–volume curve (dynamic compliance) were presented. With the interpretation of this dynamic compliance, however, several factors in the protocol should be taken into account that may have influenced the result and therefore stress a proper comparison between studies (balloon shape and characteristics, pressure- vs. volume-controlled distensions and the size of pressure or volume increments in the protocol).

In IBS, no difference was detected in compliance measured using the two distension protocols (compliance I and II), indicating that compliance can be measured in the semi-random protocol used to assess visceral perception. In healthy volunteers, however, compliance measured in the semi-random protocol resulted in higher values compared to those calculated in the staircase distension. The reason for this difference may result from the fact that healthy controls have a higher rectal compliance. The barostat device is designed to inflate or extract air from the balloon in order to maintain a certain pressure. During the semi-random staircase distension, the barostat device deflates the balloon after each pressure step, until the pressure in the balloon equals MDP. The volume at which this pressure is reached depends on the rectal tone and probably on intra-abdominal pressure. Unfortunately, we were unable to find evidence for the latter because no correlation was found between MDP and compliance. Another possible explanation for the higher volumes measured in the semi-random protocol may be that the previous distension steps from the staircase distension led to rectal adaptation and subsequent relaxation. Nozu et al. [18] reported a sensitizing effect of priming distensions in IBS patients, whereas no effect of priming on sensitivity was observed in healthy volunteers. This suggests that a difference in adaptation between healthy volunteers and IBS patients exists. We showed that compliance measurement in the semi-random protocol increases the difference between IBS patients and healthy controls and thus will help to better discriminate between those groups. An important implication of this observation is that the conventional staircase distension for measuring compliance can be discarded from barostat protocols, which results in a reduction of the duration of the total procedure by approximately 10 min per patient.

The compliance measurement is mainly used for evaluation of the pathophysiology of gastrointestinal conditions [5]. Our results show that in addition to visceral perception, compliance may also be a useful diagnostic tool and is able to discriminate between healthy controls and IBS patients.

MDP has been used in a large number of studies to correct for differences in intra-abdominal pressure between subjects [4, 5, 10, 13, 14, 19, 20]. The variation that exists between the methods to determine MDP hampers the comparison between various studies. Sometimes, MDP is reported as the pressure value at which the volume reaches 25 ml, whereas we and others defined MDP as the pressure at which respiratory waves could be detected in the balloon volume. In our opinion this method is more precise, as it allows the determination of MDP, independent of anatomical differences in the RC of the patients, although the possibility of substantial inter-observer variation should be considered when comparing different studies. In addition, the body position of the patient during MDP measurement should be considered carefully, because this greatly influences MDP. In this study, the patients were in a left lateral position to minimize the intra-abdominal pressure.

The MDP, as determined in the staircase distension, is used to correct for differences in abdominal pressure. This pressure is set to zero in the protocol for the measurement of visceral perception. A disadvantage of using the MDP as a reference is that it needs to be assessed, as well as programmed, during the actual measurement. The determination of MDP has a high inter-observer variability, which affects the accuracy of the further procedure. If MDP is set during the compliance measurement (instead of the semi-random protocol), information on the start of the pressure–volume curve will be lost because the curve will start at MDP instead of 0 mmHg. Hence, the use of MDP as a reference for barostat measurements makes the barostat technique prone to errors in conducting the measurements.

An alternative to the MDP correction could be a correction for RC. Where MDP is the balloon pressure needed to overcome the intra-abdominal pressure, RC is mostly defined as the volume at a certain pressure at the high end of the pressure range. A correlation between MDP and RC was not found. This suggests that RC, which is determined in the higher pressure range of the protocol, was influenced by other factors (such as anatomical size of the rectum or stretch of non-contractile tissue) than MDP, which is known to be affected by differences in body posture and body position.

Fox et al. [9] studied the minimal pressure at which RC should be determined with a minimal variance in the outcome measure. They showed, on the basis of results in healthy subjects, that the variance of the RC determination decreased with increasing pressure and RC should be determined preferably at a pressure of 40 mmHg. In line with these findings but limited by the maximum range of our staircase protocol, we defined a pressure of 33 mmHg to determine RC [9]. Although in the present study all IBS patients reached the pressure of 33 mmHg, the decreased pain threshold of IBS patients could potentially compromise a proper measurement of RC at higher pressure, because some patients may not complete the barostat protocol until this pressure is reached. We used a barostat balloon with a volume of 500 ml, whereas Fox et al. applied a larger balloon with a volume of 800 ml. This may have affected the pressure–volume curves due to a difference in wall tension, hampering a comparison of both studies. Within the present study though, these effects are expected to be small because none of the subjects reached the maximal balloon volume in the measurement of RC and all volunteers were measured by an identical protocol and equipment. The impact of both variables (balloon volume and pressure for RC measurement) should be studied in detail in future validation studies to reach consensus on a fully standardized procedure. On the basis of previous findings that a semi-random protocol reduces the bias that is introduced by both the predictability of the protocol and differences in tendency to report pain [21], we expect the ascending method of limits to give lower values for pain thresholds compared to phasic distensions in a random order. Conversely, Nozu et al. [18] showed that phasic distensions may sensitize IBS patients, which may result in lower pain thresholds in a semi-random protocol. We expect this sensitizing effect of the phasic distensions to be minimal in the lower volume/pressure range of the protocol because this is the first part of the assessment and only few distensions are needed to reach the value for first pain sensations. It should be noted that all subjects underwent a dummy barostat procedure after inclusion in the study, to reduce the amount of fear and anxiety on the actual day of testing and to prevent a learning curve in the consecutive test days, which may affect the study outcome.

The major advantage of correcting visceral perception data for RC, instead of MDP, is that RC correction can be done after the barostat procedure and does not require a dedicated part of the barostat protocol. This minimizes the likelihood of inaccurate measurements during the actual procedure and could reduce the procedure time by an additional 10 min. Hence, we recommend the correction for RC for visceral perception measurements. The choice of data presentation based either on pressure corrected for MDP or volume corrected for RC has implications for the individual graphs although the conclusion remained unchanged. Bouin et al. [22] previously described the sensitivity and specificity of pain thresholds in the discrimination between IBS and controls. The sensitivity and specificity to discriminate between healthy and IBS and also between sub-groups of IBS patients applying the barostat protocol as presented here will have to be assessed in follow-up studies. A cut-off score for index volume, as has been done before for pressure to discriminate between hypersensitive and normosensitive subjects, needs to be assessed.

## Conclusion

We have shown that barostat procedures in clinical practice may be shortened without losing the discriminatory value between healthy controls and IBS patients by measuring compliance during the semi-random part of the protocol, which conventionally was dedicated to assess visceral perception. The total procedure time could be shortened by 20 min to a total duration of 45 min. The exact duration of the protocol depends on the pressure step at which a patient scores the maximum sensation of pain, urge or discomfort during the perception protocol. An additional advantage of combining these measurements in the same part of the protocol may be that, when corrected for RC, the inter-observer variability may decrease. Validation of this newly proposed procedure is needed in a large group of patients in order to assess its potential and value in a clinical setting. In the near future, consensus should be reached on how to present the data (graphs vs. thresholds and volume- vs. pressure-based distensions) to enable proper comparison of different studies.

## Electronic supplementary material

Below is the link to the electronic supplementary material.

Supplementary material 1 (PDF 119 kb)
