# Supplementary material for: Alternative procedure to shorten rectal barostat procedure for the assessment of rectal compliance and visceral perception: a feasibility study
Source: J Gastroenterol. 2012 Feb 24;47(8):896–903. doi: 10.1007/s00535-012-0543-x (PMC3423561; doi:10.1007/s00535-012-0543-x)
Supplement: Supplementary file 1 — Supplementary material 1 (PDF 119 kb) [file 535_2012_543_MOESM1_ESM.pdf]

S1. Statistical analysis:

All data analyses take the experimental design into account as to provide the most appropriate analysis.

Minimal distension pressure (MDP) and first sensation (FS) data analysis:

MDP and FS were each analysed using a Gaussian linear regression ($N(\mu, \sigma^2)$ where μ is the mean and σ^2 is the variance). For both analyses, the body mass index (BMI), FS, and compliance (COMP) were included in the model.

The inference criterion used for comparing the models is their ability to predict the observed data, i.e. models are compared directly through their minimized minus log-likelihood. When the numbers of parameters in models differed, they were penalized by adding the number of estimated parameters, a form of the Akaike information criterion (AIC)[15].

For each variable of interest, a model containing the relevant covariates mentioned above ($E(y) = \beta_0 + \beta_1 \times BMI + \beta_2 \times FS + \beta_3 \times COMP$) was fitted in order to obtain a reference AIC. Then a model containing the group was fitted ($E(y) = \beta_0 + \beta_1 \times BMI + \beta_2 \times FS + \beta_3 \times COMP + \beta_4 \times Grp$).

The variable of interest was found to be differentially expressed if the AIC of the model containing a group effect was smaller than the reference AIC (the model not containing the group effect).

MDP, BMI, and rectal capacity (RC) were also analysed by bivariate Gaussian linear regression ($BVN(\mu, \Sigma)$ where μ is the mean, Σ is the two-by-two covariance matrix

$\begin{pmatrix} \sigma^2+\delta & \delta \\ \delta & \sigma^2+\delta \end{pmatrix}$, σ^2 is the variance and δ as both the extra component of variance across subjects and the common covariance among responses on the same subject) including the appropriate covariance structure in order to capture the dependence between them. The compliance and FS were included as explanatory variables. As for the previously described analysis, the AIC was used to assess a group effect.

Rectal capacity data analysis:

The RC volume was analyzed using a Gaussian non-linear regression ($\mathrm{N}(\mu, \sigma^2)$ where μ is the mean and σ^2 is the variance) including the pressure and compliance as explanatory variables. Again, the AIC was used to assess whether there was a group effect.

Visceral perception data analysis:

Pain and discomfort data were analyzed using a multivariate Gaussian non-linear regression ($\mathrm{MVN}(\mu, \Sigma)$ where μ is the mean, Σ is the covariance matrix $\begin{pmatrix} \sigma^2+\delta & \delta+\rho^{t_2-t_1} & \cdots & \text{[illegible]}^{n-t_1} \\ \delta+\rho^{t_2-t_1} & \ddots & \ddots & \vdots \\ \vdots & \ddots & \ddots & +\rho^{t_n-t_{n-1}} \\ \delta+\rho^{t_n-t_1} & \cdots & +\rho^{t_n-t_{n-1}} & \sigma^2+\delta \end{pmatrix}$, σ^2 is the variance, $\rho^{t_n-t_{n-1}}$ is the first order autocorrelation taking the time lag with the previous observation into account, and δ as both the extra component of variance across subjects and the common covariance among responses on the same subject) including, if necessary, a random effect and a first order autocorrelation.

Urge was scored on an ordinal 6-point scale and was analyzed using a mixture of a logistic distribution (parameterized as a proportional-odds) and a gamma distribution (to introduce frailty and autocorrelation dependencies)[14]. The first model was obtained by imposing the mean regression to follow a logistic ('S-shape') curve through the pressure variable ($E(y) = \beta_0 / \left(1 + e^{(\beta_1 + \beta_2 \times \text{Pressure})}\right)$). Then models including MDP and FS as explanatory variables were build ($E(y) = \beta_0 / \left(1 + e^{(\beta_1 + \beta_2 \times \text{Pressure} + \beta_3 \times \text{MDP} + \beta_4 \times \text{FS})}\right)$) in order to obtain a reference AIC. Finally, a model containing the group effect was fitted and the AIC was used to assess this group effect was significant.
